# Fibroblast activation protein alpha is expressed by chondrocytes following a pro-inflammatory stimulus and is elevated in osteoarthritis

**DOI:** 10.1186/ar1877

**Published:** 2006-01-03

**Authors:** Jennifer M Milner, Lara Kevorkian, David A Young, Debra Jones, Robin Wait, Simon T Donell, Emma Barksby, Angela M Patterson, Jim Middleton, Benjamin F Cravatt, Ian M Clark, Andrew D Rowan, Timothy E Cawston

**Affiliations:** 1 Musculoskeletal Research Group, Newcastle University, Newcastle upon Tyne, UK; 2 School of Biological Sciences, University of East Anglia, Norwich, UK; 3 Kennedy Institute of Rheumatology, Imperial College London, London, UK; 4 School of Medicine, Institute of Health, University of East Anglia, Norwich, UK; 5 Leopold Muller Arthritis Research Centre, School of Medicine, Keele University at Robert Jones and Agnes Hunt Orthopaedic Hospital, Oswestry, UK; 6 The Scripps Research Institute, La Jolla, CA 92037, USA

## Abstract

Arthritis is characterised by the proteolytic degradation of articular cartilage leading to a loss of joint function. Articular cartilage is composed of an extracellular matrix of proteoglycans and collagens. We have previously shown that serine proteinases are involved in the activation cascades leading to cartilage collagen degradation. The aim of this study was to use an active-site probe, biotinylated fluorophosphonate, to identify active serine proteinases present on the chondrocyte membrane after stimulation with the pro-inflammatory cytokines IL-1 and oncostatin M (OSM), agents that promote cartilage resorption. Fibroblast activation protein alpha (FAPα), a type II integral membrane serine proteinase, was identified on chondrocyte membranes stimulated with IL-1 and OSM. Real-time PCR analysis shows that *FAPα *gene expression is up-regulated by this cytokine combination in both isolated chondrocytes and cartilage explant cultures and is significantly higher in cartilage from OA patients compared to phenotypically normal articular cartilage. Immunohistochemistry analysis shows FAPα expression on chondrocytes in the superficial zone of OA cartilage tissues. This is the first report demonstrating the expression of active FAPα on the chondrocyte membrane and elevated levels in cartilage from OA patients. Its cell surface location and expression profile suggest that it may have an important pathological role in the cartilage turnover prevalent in arthritic diseases.

## Introduction

The proteolytic degradation of articular cartilage, leading to loss of joint function, is a major characteristic of arthritis. Cartilage consists of an extracellular matrix composed mainly of proteoglycans and collagens, in which chondrocytes, the only cell type, are embedded [1]. Degradation of proteoglycan is rapid and reversible but the breakdown of collagen is slow and essentially irreversible. Thus, collagen degradation is a key step in connective tissue breakdown.

The major extracellular proteolytic enzymes involved in cartilage resorption are the metallo- and serine proteinases, which function through a series of interacting cascades. Matrix metalloproteinases (MMPs) are zinc-dependent endopeptidases that, at neutral pH, are collectively able to degrade all components of this extracellular matrix [2]. The collagenases (MMP-1, MMP-8, MMP-13), membrane-type 1 MMP and gelatinase (MMP-2) cleave fibrillar collagen into characteristic three-quarter and one-quarter length fragments and so are key enzymes involved in cartilage collagen turnover. Cleaved collagen is unstable, unwinds and is susceptible to non-specific proteolysis. Degradation of the collagenous network is excessive in arthritis[3], and elevated levels of MMPs are detected in serum, synovial fluid, synovial membrane and cartilage from patients with arthritis [4,5]. MMPs are regulated at critical steps: synthesis, secretion, activation, inhibition, localization and clearance [6]. Activation of pro-collagenases is a crucial control point in determining if cartilage collagen resorption occurs [7]. Serine proteinases are involved in these activation cascades, although the exact serine proteinase(s) involved are not known [7,8].

Over the past few years several membrane bound serine proteinases have been identified [9]. Specific mechanisms localize proteolysis to the cell surface, which can enhance activity, limit the access of inhibitors, concentrate proteinases to their specific target substrates and limit the extent of proteolysis to discrete pericellular regions [6]. These mechanisms are important for regulating proteolytic activity. In osteoarthritis (OA), initial collagen degradation is observed around chondrocytes [3]. Thus, membrane bound MMPs and serine proteinases, as well as secreted proteinases that localize to the cell, are all important cell surface enzymes that could initiate this pericellular proteolysis. Membrane bound serine proteinases are ideally positioned to interact in these proteolytic cascades at the cell surface. The expression and characterization of membrane serine proteinases in joint tissues has not been studied and, together with the observations described above, represent an important and yet neglected area of cartilage biology.

The combination of the cytokines IL-1 and oncostatin M (OSM) added to cartilage explant cultures synergistically induces the synthesis and activation of proMMPs, leading to cartilage collagen resorption [10]. IL-1 has been shown to be involved in collagenase-mediated cleavage of collagen in OA [11]. Increased levels of both cytokines are present in the arthritic joint and adenoviral gene transfer of IL-1 in combination with OSM induces MMPs and joint damage in mice [12].

Many proteinases are regulated by complex post-transcriptional mechanisms, the understanding of which requires analysis at the protein level. Biotinylated fluorophosphonate (FP-biotin) is a rapid, specific and high-sensitivity probe enabling direct proteomic profiling of serine hydrolase activities in crude cell and tissue samples [13,14]. FP-biotin has been used previously to isolate active serine proteinases in complex proteomes. The reactivity of FP with serine proteinases requires the enzyme to be in a catalytically active state. FP-biotin binds irreversibly to serine but not cysteine, aspartate and metalloproteinases and labelled proteins are then isolated using streptavidin-agarose beads [13].

This is the first report showing the use of activity based profiling to identify active serine proteinases on chondrocyte membranes. We identify for the first time the expression of fibroblast activation protein alpha (FAPα), an integral membrane serine proteinase on chondrocyte membranes, under conditions that promote cartilage resorption and elevated expression in cartilage from OA patients.

## Materials and methods

### Materials

Recombinant human IL-1 was a generous gift from Dr Keith Ray (GlaxoSmithKline, Stevenage, UK). Recombinant human OSM was kindly donated by Professor John Heath (Department of Biochemistry, University of Birmingham, UK). FP-biotin was prepared as described previously [13,14].

### Chondrocyte membrane purification

Bovine nasal chondrocytes were isolated from nasal septum cartilage obtained from a local abattoir within 24 h of slaughter as described previously [15]. Confluent bovine nasal chondrocytes stimulated with IL-1/OSM (1/10 ng/ml) for 24 h were harvested and membrane extracts purified by sucrose-density-gradient centrifugation as described previously [5]. Membranes were resuspended in 50 mM TrisHCl pH 7.8, 0.2% v/v Triton X-100.

### Reactions between FP-biotin and chondrocyte membranes

Chondrocyte membranes (20 mg/ml in 1.5 ml reaction volume) in 50 mM TrisHCl pH 7.8, 0.14% v/v Triton X-100, 160 mM NaCl were pre-absorbed with 50 μl of streptavidin-agarose beads (Sigma-Aldrich, Poole, UK) for 1 h at 4°C with rotation. Membranes were then incubated with FP-biotin (2 μM from a 100 μM stock in dimethyl sulfoxide) for 90 minutes at room temperature with rotation. As a control for non-specific interactions, an equal amount of membranes was treated similarly but omitting FP-biotin. Labelled proteins were isolated using streptavidin-agarose beads, eluted proteins separated on 10% SDS-PAGE and then stained with colloidal Coomassie as described [14].

### Mass spectrometry

Gel bands were excised, digested in gel with trypsin and analysed by tandem electrospray mass spectrometry using a Q-Tof instrument (Waters, Manchester, UK) interfaced to a Waters CapLC capillary chromatography system as previously described [16]. Uninterpreted tandem mass spectra were searched against a database constructed by merging Swiss-Prot and TrEMBL database [17] as described [16]. Additional sequences were obtained by manual interpretation of unmatched spectra, and all deduced sequences were searched against Uniprot using the program FASTS [18].

### Chondrocyte cell culture

SW1353 human chondrosarcoma cells (ATCC, Manassas, VA, USA) were routinely cultured in DMEM containing 10% v/v fetal calf serum, 2 mM glutamine, 100 IU/ml penicillin, and 100 μg/ml streptomycin. Serum-free conditions used identical medium without fetal calf serum. For assays, cells were grown to 85% confluence and then starved of serum for 24 h before the addition of fresh serum-free medium with or without IL-1 and OSM. Experiments were performed in 12-well plates in quadruplicate. RNA was isolated from monolayers using Trizol reagent (Invitrogen, Paisley, UK).

### Real-time PCR

Total RNA (1 μg) was reverse transcribed in a 20 μl reaction using 2 μg of random hexamers and superscript II reverse transcriptase (Invitrogen) according to the manufacturer's instructions. Oligonucleotide primers were designed using Primer Express 1.0 software (Applied Biosystems, Warrington, UK). To prevent amplification of any genomic DNA present, the primers were placed within different exons close to, or spanning, the intron/exon boundary. Relative quantification of genes was performed using the ABI Prism 7900HT sequence detection system. *FAPα *expression was determined using SYBR Green (Invitrogen) using the manufacturer's suggested protocol. The primers used for human *FAPα *were: 5'-ATCTATGACCTTAGCAATGGAGAATTTGT-3' and 5'-GTTTTGATAGACATATGCTAATTTACTCCCAAC-3'. The primers used for bovine *FAPα *were 5'-ACCATGAAAAGTGTGAATGCTTCA-3' and 5'-AGTATCTCCAAAGCTTTGAATAATCACTTTCT-3'. TaqMan *GAPDH *and *18S *primers and probes were purchased from Applied Biosystems. *GAPDH *gene expression was used as an endogenous control in human cells and cartilage to normalize for differences in the amount of total RNA in each sample. In bovine samples, *18S *expression was used to normalize for differences as *GAPDH *primers and probes did not recognize bovine *GAPDH*. TaqMan mastermix reagents (Sigma-Aldrich) were used according to the manufacturer's protocol.

### Bovine nasal cartilage degradation assay

Bovine nasal cartilage explants were cultured essentially as described previously [10]. Briefly, 0.7 g of cartilage chips (approximately 2 mm in diameter by 1 to 2 mm thick) from bovine nasal septum cartilage were placed in T25 flasks and incubated overnight in 10 ml of control, serum-free medium (DMEM containing 25 mM HEPES, 2 mM glutamine, 100 μg/ml streptomycin, 100 IU/ml penicillin, 2.5 μg/ml gentamicin and 40 u/ml nystatin). Fresh control medium (10 ml) with or without IL-1 (1 ng/ml) and OSM (10 ng/ml) (each condition in triplicate) was then added (day 0). At day 7, culture supernatants were harvested and replaced with fresh medium containing the same test reagents as day 0. Cartilage and culture supernatants were harvested at days 0, 1, 3, 5, 7, 8, 10, 12 and 14 and RNA was immediately extracted from cartilage as described [5]. Hydroxyproline release was assayed as a measure of collagen degradation [10] and glycosaminoglycan release was assayed as a measure of proteoglycan degradation [10]. Collagenase activity was determined by the ^3^H-acetylated collagen diffuse fibril assay using a 96-well plate modification [19].

### Extraction of RNA from human articular cartilage

Total RNA was extracted from human articular cartilage obtained from femoral heads of patients undergoing total hip replacement surgery at the Norfolk and Norwich University Hospital as described [5]. This study was performed with Ethics Committee approval, and all patients provided informed consent. Samples from 14 patients with OA were compared with cartilage from 12 patients undergoing hip replacement following fracture of the femoral neck. OA was diagnosed by clinical history and examination along with radiographic findings; confirmation of gross pathologic findings was made at the time of joint removal. The fracture patients had no known history of joint disease and their cartilage was free of lesions. These samples are referred to herein as normal cartilage. The significance of differences between the control and OA groups was determined using a two-sided Mann-Whitney *U* test.

### Immunohistochemistry of human articular cartilage

Samples of cartilage were obtained from five patients undergoing total knee replacement for tricompartmental/end-stage OA. This study was performed with full approval from the Shropshire ethics committee. Cartilage was snap frozen in isopentene. Serial sections 10 μm thick were cut, air dried then stored at -80°C until used. Tissue sections were equilibrated to room temperature then fixed in ice cold acetone for 10 minutes. Sections were air-dried then rehydrated in PBS for 5 minutes. Endogenous peroxidase activity was blocked by incubating tissue sections in 0.3%v/v H_2_O_2 _for 15 minutes then washed for 3 × 3 minutes in PBS. Non-specific binding was blocked by incubating sections in 1.5% (v/v) horse serum in PBS for 15 minutes followed by incubation for 1 h with 10 μg/ml mouse monoclonal antibody to FAPα (Bender MedSystems, Middlesex, UK) or 10 μg/ml of a mouse IgG1 negative control (Dako, Ely, UK). Antibody binding was detected and visualised using horseradish peroxidase Vectastain ABC Elite kit (Vector Laboratories, Peterborough, UK) followed by a 3,3'- diaminobenzidine/nickel staining kit (Vector Laboratories). Sections were then counterstained with Mayer's hematoxylin solution (Sigma-Aldrich).

## Results

### Identification of FAPα in chondrocyte membrane extracts

**Figure 1:**
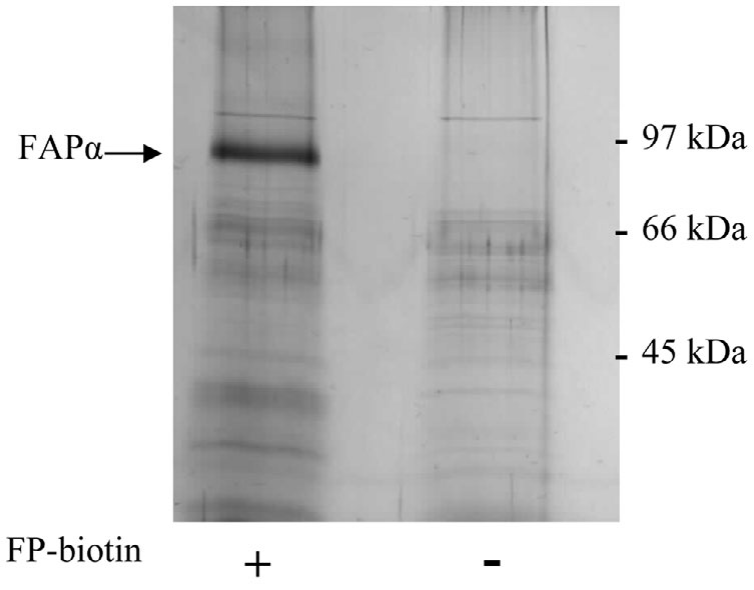
Biotinylated fluorophosphonate (FP-biotin) labelling of fibroblast activation protein alpha (FAPα) on chondrocyte membranes. Membrane extracts isolated from IL-1 plus oncostatin M stimulated chondrocytes were treated with or without FP-biotin. Labelled proteinases were isolated using streptavidin-agarose beads and eluted with reducing SDS-PAGE loading buffer. Proteins were separated by SDS-PAGE and stained with colloidal Coomassie. The 97 kDa protein was identified by mass spectrometry to be FAPα.

In OA, initial collagen degradation is observed around the pericellular region surrounding the chondrocyte [3]. Thus, membrane proteinases are ideally positioned to interact in pericellular proteolysis. FP binds irreversibly to active serine proteinases; therefore, we have used FP-biotin to probe chondrocyte membranes for serine proteinase activities. We have previously shown that the addition of IL-1 plus OSM to cartilage explant cultures results in cartilage resorption [10]. To identify serine proteinases synthesized by chondrocytes under these conditions, bovine nasal cartilage chondrocytes were stimulated with IL-1 plus OSM for 24 h. The use of bovine cells enabled large-scale preparation of membranes for protein identification by mass spectrometry. Following incubation of chondrocyte membranes with FP-biotin, a major band was observed at approximately 97 kDa (Figure 1). This was not detected in membranes incubated in the absence of FP-biotin, confirming specificity. Tandem mass spectrometry enabled sequencing of 11 peptides (162 amino acid residues: Table 1) from the tryptic digest of the 97 kDa band. When searched against the Uniprot database using FASTS [18], these deduced amino acid sequences matched human FAPα (Uniprot ID:Q12884) with 95% identity (the bovine ortholog is not yet present in any publicly available protein database). The slight divergence from the human sequence (for example, deletion of G143; Table 1) was comparable to that between human and mouse FAPα. Thus, probing the chondrocyte membrane with FP-biotin has identified active FAPα.

### Regulation of *FAPα *gene expression in chondrocytes

**Figure 2:**
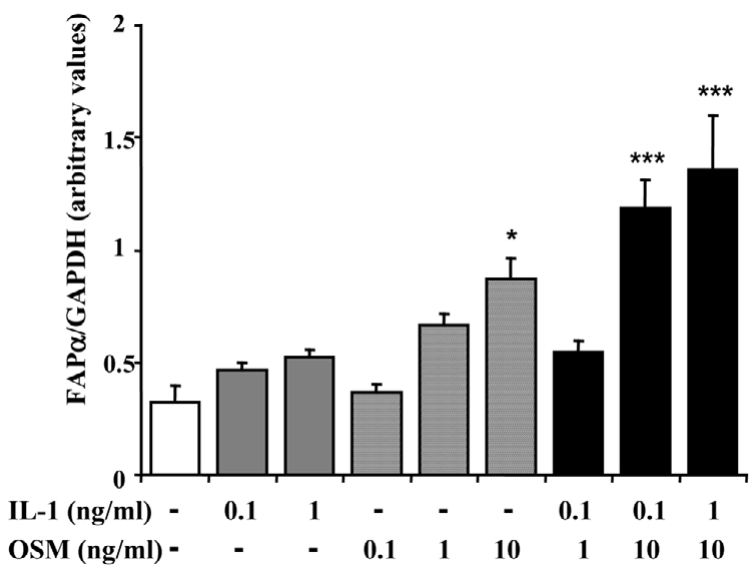
*FAPα *gene expression is upregulated by IL-1 and oncostatin M (OSM) in chondrocytes. SW1353 cells were treated with combinations of IL-1 and OSM for 24 h. Total RNA was extracted and *FAPα *gene expression determined by real-time PCR as described in Materials and methods. The data are presented relative to *GAPDH*, and are representative of four separate experiments. * = *P *< 0.05, *** = *P *< 0.001 versus control

The regulation of FAPα gene expression by IL-1 and OSM, cytokines known to promote cartilage resorption, was investigated in the SW1353 chondrocytes using real-time PCR (Figure 2). IL-1 alone induces low levels of FAPα gene expression and OSM alone induces higher expression, while the combination of IL-1 and OSM further increases FAPα expression. Thus, FAPα expression is up-regulated under conditions that promote cartilage resorption.

### Regulation of *FAPα *gene expression in resorbing cartilage

**Figure 3:**
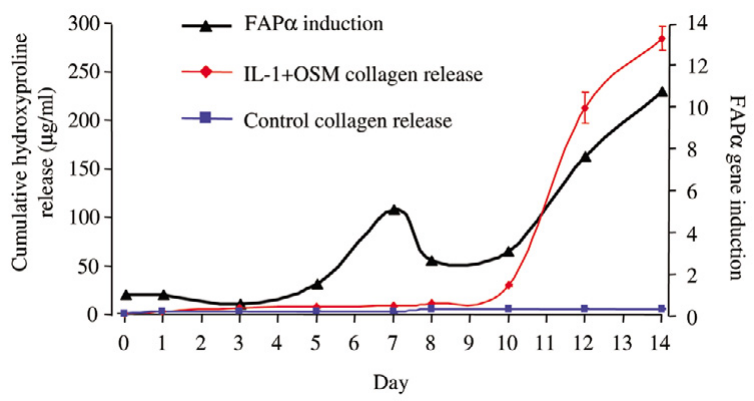
*FAPα *gene expression is induced in resorbing cartilage. Bovine nasal cartilage chips were cultured in medium with or without IL-1 (1 ng/ml) and oncostatin M (OSM; 10 ng/ml) for 14 days. At day 7, medium was removed and the cartilage was replenished with identical reagents. Cartilage and medium were harvested at days 0, 1, 3, 5, 7, 8, 10, 12 and 14. Each time-point and condition were performed in triplicate. As a measure of collagen, the levels of hydroxyproline released into the media from unstimulated (control) and IL-1/OSM stimulated cartilage were assayed and cumulative hydroxyproline release is shown. Values are the mean ± standard error of the mean. RNA was extracted from cartilage and *FAPα *gene expression was determined by real-time PCR as described in Materials and methods. The data are presented relative to *18S *and show fold induction of *FAPα *by IL-1/OSM compared to control treatments.

Collagenases degrade cartilage collagen and our previous work has shown that serine proteinases are involved in the cascades leading to activation of these pro-collagenases [7,8]. To determine if FAPα is expressed in resorbing cartilage, the expression of *FAPα *was investigated in an IL-1 plus OSM induced bovine nasal cartilage degradation assay (Figure 3). IL-1 plus OSM induces a rapid breakdown of proteoglycan, with over 80% release by day 5 of culture (data not shown). Active collagenase is first detected at day 10 of culture (data not shown), followed by a rapid release of collagen fragments (Figure 3). *FAPα *gene expression is significantly induced at days 7 to 14 of culture (Figure 3). The lower level of *FAPα *induction seen at days 8 to 9 is likely due to the effects of changing the medium and re-stimulating the cartilage with cytokines at day 7. *FAPα *gene expression is induced in resorbing cartilage after proteoglycan release, but prior to and during collagen release, thus suggesting that FAPα could be associated with the mechanisms leading to cartilage collagen degradation.

### *FAPα *gene expression in human articular cartilage

**Figure 4:**
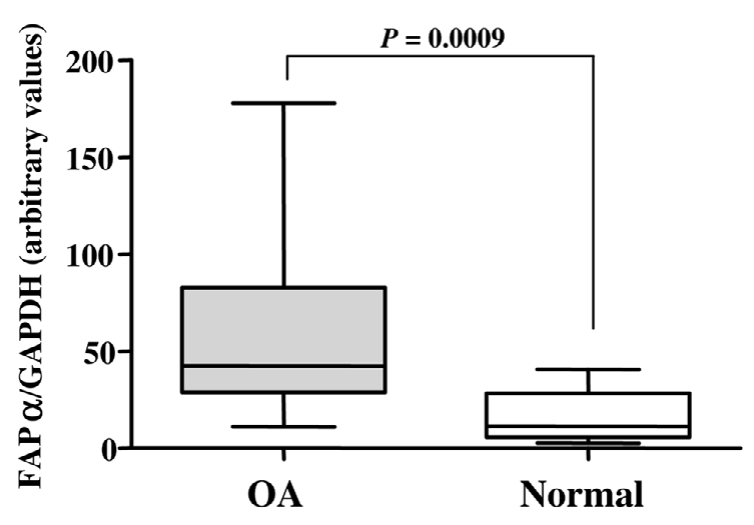
*FAPα *gene expression is upregulated in osteoarthritic cartilage. Total RNA was extracted from osteoarthritic hip cartilage (n = 14) and phenotypically normal hip cartilage from patients with femoral neck fracture (n = 12). *FAPα *gene expression was determined by real-time PCR as described in Materials and methods. The data are presented relative to *GAPDH*. *FAPα *gene expression is significantly higher in osteoarthritic cartilage (OA) compared to normal cartilage (*P *= 0.0009).

To evaluate the expression of FAPα in arthritic disease, the levels of FAPαgene expression were compared in normal and osteoarthritic cartilage (Figure 4). FAPα gene expression is significantly higher in osteoarthritic (mean = 61.1) compared to normal (mean = 16.1) cartilage (*P* = 0.0009).

### Immunohistochemistry analysis of FAPα in cartilage from OA patients

**Figure 5:**
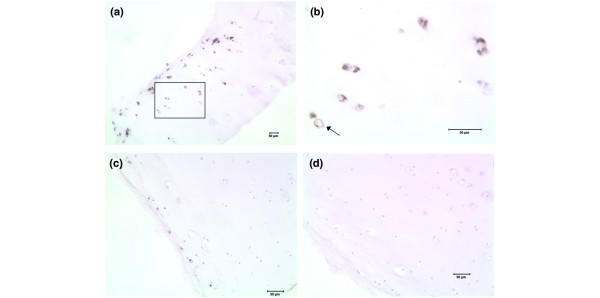
Immunolocalisation of FAPα protein in osteoarthritic (OA) cartilage. **(a) **FAPα in OA cartilage specimen 1. Note positive staining (brown/black) of cells in the superficial zone. Boxed region represents low-power view of Figure 5b. **(b) **High-power view of FAPα in OA cartilage specimen 1. Note positive staining of the chondrocyte membrane (arrow). **(c) **FAPα in OA cartilage specimen 2. Note positive staining of cells in the superficial zone. **(d) **OA cartilage specimen 2 treated with non-immune mouse IgG as a negative control for FAPα.

Immunodetection of FAPα was demonstrated in all cartilage sections from OA patients examined (n = 5). Staining was observed in the superficial zone (Figure 5a,c) and on the chondrocyte membrane (Fig. 5b). No immunostaining was observed in OA cartilage treated with a negative control non-immune mouse IgG (Figure 5d).

## Discussion

We report for the first time the use of activity-based probes to identify proteinases in resorbing cartilage. This is the first study to show that chondrocytes synthesize FAPα (also known as seprase) when stimulated with the pro-inflammatory cytokines IL-1 and OSM. *FAPα *gene expression is induced just prior to collagen degradation in a model of cartilage resorption, thus suggesting that FAPα is associated with collagen resorption. Furthermore, *FAPα *gene expression is significantly elevated in cartilage from patients with OA. Immunohistochemistry analysis shows staining for FAPα on chondrocytes in the superficial zone of OA cartilage. In OA, the superficial zone is characterised by fibrillations and degenerative matrix changes, and proteinases involved in cartilage resorption, such as the collagenases (MMP-1, MMP-8 and MMP-13), also show highest expression in the superficial zone of OA cartilage [20]. These observations support a role for FAPα in the mechanisms leading to cartilage degeneration in OA.

FAPα was initially identified as a cell surface glycoprotein present on stromal fibroblasts of human epithelial cancers [21] and on the invadopodia of a human malignant melanoma cell line LOX, which exhibits aggressive behaviour in experimental metastasis [22,23]. Immunohistochemistry has shown that FAPα is transiently expressed in certain normal fetal mesenchymal tissues, but in normal adult tissues FAPα expression is absent. Most of the common types of epithelial cancers, including over 90% of breast, lung and colorectal carcinomas, contain abundant FAPα expression. It is strongly expressed by the reactive tumour stromal fibroblasts surrounding the newly formed blood vessels of epithelial cancers and in reactive fibroblasts found in the granulation tissue of healing wounds [21]. FAPα is also expressed by stellate cells at the tissue remodelling interface in human cirrhosis but not in normal livers [24]. Thus, FAPα is expressed in many pathologies.

FAPα is a type II transmembrane serine proteinase with a cytoplasmic tail that contains six amino acids followed by a 20 amino acid transmembrane domain at the amino terminus, a region with several potential *N*-glycosylation sites, a cysteine rich substrate-binding domain and a stretch of 200 amino acids at the carboxyl terminus containing the catalytic serine, aspartate and histidine in a non-classical orientation [25,26]. The active enzyme is a homodimer that contains two 97 kDa subunits [27]. FAPα is structurally very similar to dipeptidyl peptidase IV (DPPIV). Both enzymes have dipeptidyl peptidase activity and cleave prolyl peptide bonds (Pro-Xaa). DPPIV has a variety of known substrates, including chemokines, growth factors, neuropeptides and vasoactive peptides; however, the natural ligand of FAPα is not known. FAPα has been shown to have both exo- and endopeptidase activity and can cleave gelatin [24,28]. Thus, during cartilage resorption FAPα may contribute to the degradation of denatured collagen (gelatin) after the initial cleavage by collagenases. FAPα associates with DPPIV, MMP-2, membrane-type 1 MMP and urokinase plasminogen activator receptor at invadopodia of human malignant melanoma cells [22,29] and so may interact with these proteinases and receptors and associated cascades. For example, DPPIV and FAPα can form a complex localised at invadopodia of fibroblasts on collagenous fibres that has both gelatinolytic and gelatin binding activities, which allow cell migration [30].

In a murine collagen-induced arthritis model, gene-expression profiling using the Mu11K array (Affymetrix) showed a seven- fold increase in *FAPα *gene expression together with MMP expression in inflamed, compared to non-inflamed, paws [31]. In addition, *FAPα *maps to a chromosomal region containing collagen-induced arthritis linked susceptibility loci [32], which is also consistent with a role in arthritis. The *FAPα *ortholog in *Xenopus laevis *has been reported to be induced during tadpole metamorphosis [33], a process intimately associated with collagenolysis. The first collagenase enzyme was isolated from such tissue [34]. Furthermore, *FAPα *is also induced at regions of active tissue remodelling during mouse embryogenesis, including somites and perichondrial mesenchyme from cartilage primordia [35].

These data and our own observations clearly support a role for FAPα in tissue remodelling processes during normal development and in pathology. Further studies are required to determine the exact mechanistic role of FAPα in tissue proteolysis.

## Conclusion

Using an active-site probe, we have identified for the first time active FAPα, a serine proteinase on the chondrocyte membrane. We have shown that *FAPα *gene expression is up-regulated by pro-inflammatory cytokines IL-1 and OSM in chondrocytes and is induced during cartilage collagen resorption. Furthermore *FAPα *gene expression is significantly elevated in cartilage from OA patients when compared to age-matched normal controls. Immunohistochemistry analysis of cartilage from OA patients shows FAPα staining on chondrocytes in the superficial zone. Although the exact function of FAPα remains to be elucidated, we clearly show an association between FAPα and chondrocytes in the context of cartilage degradation. The surface location of FAPα ideally positions it for a role in pathological pericellular tissue degradation and remodelling in cartilage as is seen in arthritic diseases.

## Abbreviations

DMEM = Dulbecco's modified Eagle's medium; DPPIV = dipeptidyl peptidase IV; FAPα = fibroblast activation protein alpha; FP-biotin = biotinylated fluorophosphonate; IL = interleukin; MMP = matrix metalloproteinase; OA = osteoarthritis; OSM = oncostatin M; PBS = phosphate buffered saline; PCR = polymerase chain reaction.

## Competing interests

The authors declare that they have no competing interests.

## Authors' contributions

JMM helped conceive, design and coordinate the study and carried out membrane preparations, FP-biotin experiments, preparation of cDNA from bovine cartilage, real-time PCR, immunohistochemistry and drafted the manuscript. LK prepared cDNA from cartilage. DAY coordinated human cartilage collection and cDNA preparation and helped with preparation of cDNA from bovine cartilage. DJ carried out tissue culture. RW carried out the mass spectrometry. BFC prepared the FP-biotin. STD collected cartilage from joint replacement surgery. IMC coordinated human cartilage collection and cDNA preparation. EB prepared cartilage sections for immunohistochemistry. AMP collected and prepared cartilage for immunohistochemistry. JM coordinated collection of OA cartilage for immunohistochemistry. ADR helped to conceive, design and coordinate the study and draft the manuscript. TEC helped to conceive, design and coordinate the study and draft the manuscript. All authors read and approved the final manuscript.

## Figures and Tables

**Table 1:** Peptide sequence confirmation of bovine fibroblast activation protein alpha from chondrocyte membranes.

*m/z *(Charge)	Location^a^	Sequence
564.78 (2+)	335–343	TQEHIEESR
736.77 (2+)	367–375	IFSDKDGYK
611.30 (3+)	210–219	YALWWSPNGK
751.41 (2+)	162–173	LAYVYQNNIYLK
796.41 (2+)	592–605	LGVYEVEDQITAVR
969.56 (2+)	534–550	YPLLIQVYGGPCSQSVR
697.41 (2+)	510–521	LKVDDITLWYK
786.47 (2+)	551–564	SIFAVSWISYLASK
715.65 (3+)	144–161	NELPRPIQYLCWSPVGSK
776.36 (3+)	124–142	YSYTATYHIYDLTNGEFIR
1098.96 (2+)	403–421	VTQDSLFYSSNEFEGYPGR

The 97 kDa band obtained following biotinylated fluorophosphonate labelling of bovine chondrocytes was trypsin digested and analysed using mass spectrometry; 11 separate peptides were detected. ^a^Numbering according to uniprot entry Sepr_Human. Human has Y not H in position 131, G143 is deleted in the bovine sequence, human has E not G in position 417, E not K at 511, E not D at 514, V not I at 552, and N not S at 556.
